# The Controlled Cortical Impact Model: Applications, Considerations for Researchers, and Future Directions

**DOI:** 10.3389/fneur.2016.00134

**Published:** 2016-08-17

**Authors:** Nicole D. Osier, C. Edward Dixon

**Affiliations:** ^1^Department of Acute and Tertiary Care, University of Pittsburgh School of Nursing, Pittsburgh, PA, USA; ^2^Safar Center for Resuscitation Research, Pittsburgh, PA, USA; ^3^Department of Neurosurgery, University of Pittsburgh School of Medicine, Pittsburgh, PA, USA; ^4^VA Pittsburgh Healthcare System, Pittsburgh, PA, USA

**Keywords:** controlled cortical impact, experimental brain injury, brain trauma, pre-clinical, review

## Abstract

Controlled cortical impact (CCI) is a mechanical model of traumatic brain injury (TBI) that was developed nearly 30 years ago with the goal of creating a testing platform to determine the biomechanical properties of brain tissue exposed to direct mechanical deformation. Initially used to model TBIs produced by automotive crashes, the CCI model rapidly transformed into a standardized technique to study TBI mechanisms and evaluate therapies. CCI is most commonly produced using a device that rapidly accelerates a rod to impact the surgically exposed cortical dural surface. The tip of the rod can be varied in size and geometry to accommodate scalability to difference species. Typically, the rod is actuated by a pneumatic piston or electromagnetic actuator. With some limits, CCI devices can control the velocity, depth, duration, and site of impact. The CCI model produces morphologic and cerebrovascular injury responses that resemble certain aspects of human TBI. Commonly observed are graded histologic and axonal derangements, disruption of the blood–brain barrier, subdural and intra-parenchymal hematoma, edema, inflammation, and alterations in cerebral blood flow. The CCI model also produces neurobehavioral and cognitive impairments similar to those observed clinically. In contrast to other TBI models, the CCI device induces a significantly pronounced cortical contusion, but is limited in the extent to which it models the diffuse effects of TBI; a related limitation is that not all clinical TBI cases are characterized by a contusion. Another perceived limitation is that a non-clinically relevant craniotomy is performed. Biomechanically, this is irrelevant at the tissue level. However, craniotomies are not atraumatic and the effects of surgery should be controlled by including surgical sham control groups. CCI devices have also been successfully used to impact closed skulls to study mild and repetitive TBI. Future directions for CCI research surround continued refinements to the model through technical improvements in the devices (e.g., minimizing mechanical sources of variation). Like all TBI models, publications should report key injury parameters as outlined in the NIH common data elements (CDEs) for pre-clinical TBI.

## Introduction and Purpose

Traumatic brain injury (TBI) is a significant worldwide public health problem (1–7). Individuals who survive TBI often require extensive care, including immediate and emergent care often followed by extensive rehabilitation; taken together, this care is associated with high direct and indirect costs (8). Unfortunately, to date, efforts to develop therapies effective at improving outcomes of clinical TBI have fallen short and not led to novel FDA-approved therapies for TBI patients. Continued research is needed, including the utilization of experimental (i.e., pre-clinical) TBI models before attempts to translate interventions to the clinical setting can be justified.

One of the most widely used models of experimental TBI is controlled cortical impact (CCI). Developed in the 1980s, the CCI model has been adapted and refined extensively in the years since. The purpose of this manuscript is to provide a primer on the past and current applications of CCI and discuss considerations for the future of CCI research. In doing so, this review will: (1) provide an overview of the CCI model, (2) synopsize the history of the model’s development, (3) highlight the models strengths and weaknesses, (4) discuss experimental design considerations for researchers using CCI, and (5) identify future directions for CCI research along with ways to improve the model. When relevant, details regarding information that should be reported for CCI studies based on the National Institute of Neurological Diseases and Stroke (NINDS) pre-clinical common data elements (CDEs) will be noted.

## Past and Present Applications: Model Overview, Development, Features, Subtypes, and Areas of Inquiry

### Overview of the CCI Model

The CCI model was developed in the late 1980s and rapidly became one of the most commonly used models of pre-clinical TBI. The early devices were pneumatically driven, and more recently electromagnetic devices have become available. In the following sections, the development of CCI will be summarized along with notable applications to date, the key features of CCI will be described, and the different devices (pneumatic; electromagnetic) will be discussed.

#### Model Development and Key Applications

For over a century, efforts to understand human TBI have relied on utilization of animal models (9–11) to supplement clinical evidence. Early studies were characterized by small sample sizes, a lack of well-established (i.e., vetted) devices for injury induction, and low levels of control over potential confounders. Starting in the 1970s, efforts to refine, standardize, and quantify experimental brain injury models became increasingly common (12–18). Contemporary CCI models owe their lineage to the work of Thomas Anderson (19, 20), who was the first to develop a neurotrauma model utilizing a constrained-stroke pneumatic cylinder mounted on an adjustable crosshead frame to produce injury with a high degree of mechanical reproducibility. The CCI model was first developed in the late 1980s and early 1990s by Lighthall and colleagues to induce TBI in ferrets (21, 22). By 1991, the device was adapted so that the model could be applied to rats (23). After translation to rats, CCI has since been applied to mice (24–26), swine (27–30), and non-human primates (31), as described in detail later in this review.

Following the initial development and characterization of the model, several new applications of CCI device have emerged, including options for studying closed head injury (CHI) (32–34). The first three decades of CCI research shows some progression in the types of research questions addressed. Early applications primarily focused on characterizing the model and exploring the biomechanical and physiological changes associated with injury. Later efforts expanded the histopathological and cellular characterization using brain tissue after CCI to identify putative secondary injury processes that could not easily or ethically be studied in human TBI survivors. CCI has also been used extensively to test novel therapies in the hope of ultimately translating promising drugs to clinical care (35–38).

More recently, the expansion of transgenic animals has led to applications of CCI to identify important genes and gene products that impact injury severity and recovery profiles (39–42). Notably, the trend is not linear and ongoing research is being conducted in all of the aforementioned areas, with efforts to increasingly promote rigor and replication through the use of CDEs. Today, CCI remains a mainstay in pre-clinical TBI research.

#### Key Features of the Model

Traditionally, CCI is a mechanical model of TBI that follows anesthetized craniectomy. The CCI device mechanically transfers energy onto the intact dura mater damaging the cortex, and sometimes the subcortical structures in cases of more severe injury. Applications of CCI are discussed in more detail elsewhere in this review. A key feature of CCI is that the injury parameters (e.g., depth, velocity, and dwell time) can all be controlled for to produce a broad range of TBI severities and well as closed head impact by impacting the intact skull.

#### Device Types and Suppliers

Two main types of CCI devices are commercially available: pneumatic and electromagnetic. The original applications of the CCI model used a pneumatic device and pneumatic CCI is still commonly used today. The electromagnetic alternative was developed later but is gaining popularity due to its lower cost and greater portability. Both types of CCI are discussed in detail below, including a list of commercial suppliers (Table 1).

**Table 1:** **Summary of CCI types and commercial suppliers**.

	Company (alphabetical)	Location	Device	Notes/comments
Electromagnetic	Hatteras Instruments	Cary, NC, USA	Pinpoint PCI3000 Precision Cortical Impactor	– Removable tips (seven sizes available) – Three system configurations – Suitable for large animal models when used with articulated support arm (accessory unit)
	Leica Biosystems	Buffalo Grove, IL, USA	Impact One Stereotaxic Impactor for CCI	– Removable tips (comes with 1-, 1.5-, 2-, 3-, and 5-mm tips)
Pneumatic	Amscien Instruments	Richmond, VA, USA	Pneumatic (Cortical) Impact Device (Model: AMS 201)	– Accessory unit to measure rod speed is also available
	Precision Instruments & Instrumentation, LLC	Lexington, KY, USA	TBI-0310 Impactor	– Removable tip (3 and 5 mm standard) – Custom tips for sale

##### Pneumatic

**Figure 1:**
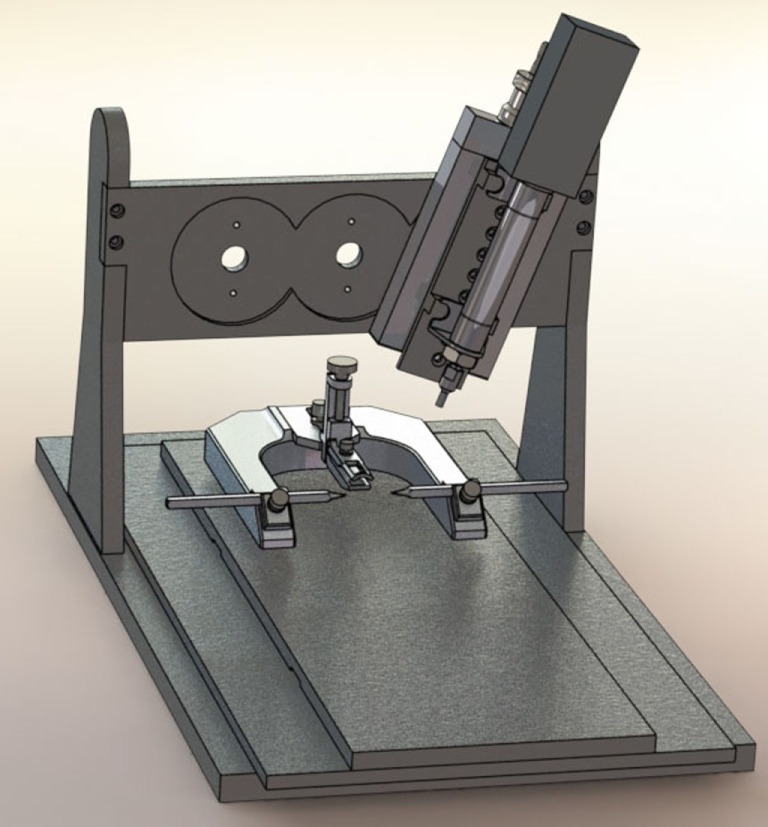
**Pneumatic controlled cortical impact device (Pittsburgh Precision Instruments, Pittsburgh, PA, USA)**.

When Lighthall and his colleagues first developed the CCI model, the device was powered by pressurized gas (i.e., pneumatically driven). Pneumatic CCI remains widely used to study TBI pathophysiology and test novel therapies (43–45). A typical pneumatic CCI device (Figure 1) includes a cylinder, which is rigidly mounted to a crossbar. There typically are multiple mounting positions on the crossbar so the impactor can be vertical or angled, respective to the skull and underlying brain tissue. Pneumatic CCI devices have a small-bore reciprocating double-acting pneumatic piston with a maximum adjustable stroke length of approximately 50 mm. This piston functions to propel a tip into the exposed neural tissue or, in the case of CHI models, the intact skull (discussed elsewhere in this review). Depending on the research goals, tips of varied sizes and shapes are available, as described later.

##### Electromagnetic

**Figure 2:**
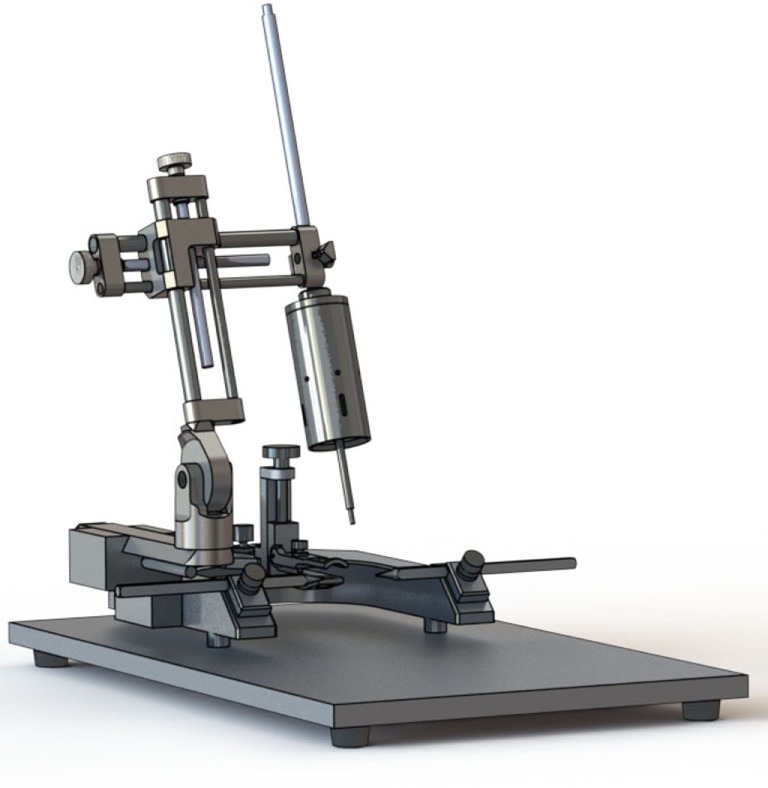
**Electromagnetic controlled cortical impact device (Leica Biosystems, Buffalo Grove, IL, USA)**.

More recently, electromagnetic CCI devices have become available and they share many features with pneumatic devices (Figure 2). As with the pneumatic device, the electromagnetic alternative is traditionally used in combination with a commercial stereotaxic frame, facilitating adjustment of the impactor angle. Some devices are also compatible with an articulated support arm that can elevate the injury device to facilitate modeling CCI in swine and other large animals. The electromagnetic device is potentially more portable than pneumatic devices due to its smaller size and ability to function without a pressurized gas source. A number of options for tip size and shape are also available. Like the pneumatic CCI devices, the electromagnetic devices are also considered to create a reproducible model of brain trauma. Notably, there is little empirical evidence comparing the pneumatic and electromagnetic models; however, one study suggested greater reproducibility with electromagnetic CCI compared to pneumatic CCI (46).

##### Commercial Suppliers

Over the last decade, several CCI devices have become available from commercial suppliers (Table 1). An advantage is that CCI standardization is increased. For each category of CCI, the company names, locations, and devices are provided. Additional details are also provided, including information about the available tips and accessory units.

### Diverse Applications and Other Advantages of the CCI Model

Controlled cortical impact has many strengths and, consequently, the model has had many applications. Below is a brief overview of some of the key strengths of the model. Among the topics included are the use of CCI in many species of test subject, the clinical relevance of the model, scalability, and control over important injury parameters.

#### Control

A key strength of CCI is the high degree of control over mechanical factors that may affect study findings, including impact depth, velocity, dwell time, and volumetric characteristics associated with varying impact tip sizes. Additional detail regarding how to make decisions about these injury parameters are provided later in this review (see Impactor Characteristics: Size, Shape, Depth, Velocity, Dwell Time, Angle, and Composition). Beyond the CCI parameters, there are general experimental conditions that can be controlled, including housing, husbandry, diet, light/dark cycles, anesthesia (e.g., type; dose; duration), and other factors. While physiological and other confounding factors are not as tightly controllable, they can be monitored and data recorded, including temperature, respiration rate, and light/dark cycle. Provided the laboratory is equipped with the necessary and properly maintained and calibrated equipment (e.g., heating pad; temperature probe; ventilation set up), control over these parameters adds little additional burden to the researcher. Details surrounding variables controlled in CCI studies should be reported in accordance with recommended pre-clinical common data elements (47).

#### Appropriate for Use in Multiple Species

Another key strength of the CCI model is its scalability, resulting in the use of the CCI model in multiple species of test animals. For example, CCI has been used in ferrets (21, 22), rats (23, 48, 49), mice (25, 50–52), swine (27, 30, 53), and non-human primates (31). A summary of species used in CCI research is provided below (Table 2) along with an example of how injury parameters have been set in published studies, though variability across studies exists within a single test species. When scaling injury parameters across species, a recommended starting point is to normalize the percent of brain volume deformed relative to the total brain volume. Beyond scaling the injury parameters, additional modifications to the device may be necessary; for example, the impactor may need to be mounted to an appropriately sized frame or used in combination with an articulated support arm in order to position the tip above the head of a larger animal.

**Table 2:** **Summary of species CCI has been used and examples of injury parameters**.

Animal (alphabetical)	Injury site	Depth (mm)	Dwell time (ms)	Velocity (ms)	Craniectomy size	Tip diameter (mm)
Mouse	Parietal cortex	0.5–2	50–250	4–6	4–5 mm	3
Rat	Parietal cortex; midline	1–3	50–250	4	6–8 mm	5–6
Pig	Frontal lobe	12	50–400	2–4	16–18 mm	15
Primate	Frontal lobe	7	150	3.5	11–12 mm	10

#### Clinical Relevance

Overall, CCI is considered a clinically relevant pre-clinical TBI model by virtue of reproducing many aspects of clinical TBI. Topics covered in this review include the pathophysiological and functional consequences produced that are similar to what is seen clinically, the ability to model TBI across the lifespan, as well as applications for evaluating repeated head injury and testing novel therapies.

#### Pathophysiological Consequences That Mimic TBI Pathophysiology

One key component of the clinical relevance of the CCI model is its ability to produce many of the histopathological changes seen in TBI patients. Gross histological changes known to follow CCI include: cortical contusion (23, 54–56), disruption of the blood–brain-barrier (57, 58), hippocampal cell loss (59–63), and overall brain volume loss (64–66). Furthermore, clinical injury is characterized by many secondary injury cascades that have also been reported as activated after CCI, as evidenced by histological markers of several processes, including apoptosis (67–71), inflammation (64, 71–74), and oxidative stress (74–79). Notably, many of the pathophysiological consequences of TBI have been found to persist into the chronic period, defined in a recent review as persisting at least 2 weeks following experimental TBI (80). For example, CCI is known to result in chronic ventricular enlargement and shrinkage of gray and white matter (65, 81), apoptosis (69, 82, 83), necrosis (24, 84), axonal injury (85), and inflammation (86).

#### Functional Deficits That Model Symptoms Experienced by Survivors

Adding to the clinical relevance of CCI is the functional deficits in many domains, including overall neurological function (23, 87, 88), memory (24, 65, 89), learning (90, 91), motor function (92–94), and frontal lobe function (95–97). In rodent models, these outcomes can be readily assessed using reliable and valid tests, including the Morris water maze (65, 90), Barnes maze (25, 98), elevated plus maze (63), rotarod (99, 100), beam balance task (23, 92), beam walking task (23, 24, 87, 93, 98), and attentional set-shifting test (AST) (101), to name a few. While standardized assessments of functional outcomes are better established in small mammals, there are also options for functional outcome testing in large mammals, though there remains a relative lack of normative data compared to rodents (28). Similar to the pathophysiological consequences of CCI, functional deficits often persist into the chronic period (102). For example, chronic deficits have been reported in frontal lobe dysfunction (95), overall neurological function (87, 103–105), motor function (87, 93, 98, 106–110), and cognitive function (50, 87–89, 92, 93, 103, 104, 111–116).

#### Appropriate for Use in Long-Term Survival Studies and in Test Animals of All Ages

The CCI model is commonly used and has been used in over 1000 PubMed-indexed manuscripts. In addition to the aforementioned assets of the model, CCI has a low mortality rate, relative to the fluid percussion model. The high survivability of CCI is beneficial in practical ways: it reduces sample attrition, keeps study costs down, and assists in the effort to minimize animal suffering. CCI’s low mortality rate also facilitates studying the chronic effects of TBI. Notably, there is ongoing discussion surrounding how long after the initial injury functional impairments, and the underlying pathology, persist. To date, no study has followed the natural history of CCI from the time of the initial injury to spontaneous death; rather, all published studies sacrifice test animals at a controlled time point. In preparing this paper, the longest data collection period after CCI identified in published papers was 1-year post-injury (65, 117, 118). Interestingly, several pathophysiological changes were reported at 1 year after CCI induction, including chronic microglial activation (117), lesion volume expansion (65, 117), hippocampal neurodegeneration (117), ventricular enlargement (65), myelin loss (117), and compensatory responses in cholinergic neurons (65). Only one CCI study reported functional deficits a year after TBI, specifically poorer performance on the Morris water maze hidden platform task and probe trial (65).

The scalability of the CCI model also make it appropriate for use in test animals of any age. For example, CCI is commonly used model for studying the effects of injury on the immature brain as it can be easily scaled down by using a smaller tip and driving the tip less deep into the brain tissue (27–29, 48, 119–121). Similarly, CCI has been used to test outcomes of injury later in life, including using mice prone to senescence (122–125). Still, most CCI studies use adult male rodents as the subjects. Rationale for most studies using male animals is that the majority of TBI patients in every age group are male (2), and female hormones may influence TBI outcomes (126). However, several CCI studies have had samples comprised entirely or partly of female test animals (127–130).

#### Ability to Test the Effects of Closed Head Injury and Repetitive Injury

CHI is an additional research application of the CCI device (111). A primary advantage to using a CCI impactor over weight drop methods is that a higher velocity of the impact can be achieved (23). CCI devices have been used to impact the exposed skull (46), intact scalp (33), and “helmeted” head (131). Frequently, the procedures are set up to allow for some degree of head motion after impact. Beyond studying the effects of single CHI, CCI is also used to test the effects of repetitive impacts (29, 33, 34, 66). The effects of CHI and repetitive injury are relevant to many clinical populations, including athletes, military personnel, and victims of domestic violence. Pneumatic impactors have also been used to create sports-related head rotational acceleration in rats by striking a laterally extended arm of a helmet (132). Repetitive injuries using the CCI device are associated with worse functional outcomes, when compared to a single concussion (33). Currently, the translatability of these CHI models to clinical settings is gauged by similarities of the pathological responses. For example, CCI–CHI models have been reported to produce alterations in clinically relevant cognitive, cerebrovascular, and morphological domains; as well as symptoms related to memory; emotion; and sleep (33, 81, 133).

#### Testing Therapies

Despite the wide variety of therapies that have shown benefit in pre-clinical trials, including CCI, none have resulted in an FDA-approved therapy for TBI in the United States. Thus, there remains an impetus to identify promising therapies in pre-clinical models and translate them to clinical trials and ultimately clinical care (37, 134). In this effort, CCI represents an important and commonly used model; evidence from CCI studies in combination with other models has led to identification of therapies for clinical trials, though the success of these trials has been limited. For example, edaravone’s effects and therapeutic window were tested in a TBI study (38) and later applied to clinical care of TBI in Japan. In the United States, both pre-clinical studies and clinical trials of amantadine have shown promise in promoting neurobehavioral recovery after TBI (35, 36, 135, 136). It is also worth noting that several novel therapies (e.g., hypothermia; progesterone; cyclosporine) had successful phase II trials but were unable to withstand phase III trials (137–139). Some drugs that had been used clinically for symptom management were tested for their effects on TBI outcomes in studies using CCI and other pre-clinical models, including methylphenidate (140–143), amantadine (35), and levetiracetam (144–146). Thus, translation between pre-clinical and clinical trials of TBI therapeutics is bidirectional.

## Limitations of the CCI Model and Potential Alternatives

Though CCI has many notable strengths, as described above, there are some limitations associated with the model. Notably, many of these limitations are common to many or all pre-clinical TBI models. Only a few limitations are unique to CCI. Later in the review, considerations for researchers regarding how to minimize these concerns via thoughtful experimental design will be extolled.

### Concerns Common to Many Pre-Clinical Models

The CCI model shares many of the same limitations as other common pre-clinical TBI models, because some experimental conditions differ substantially from the experiences of TBI patients. Being anesthetized at the time of injury may confound study findings, though these effects are mitigated by the use of sham controls who also receive anesthesia and other identical treatment excluding the injury itself. Concerns regarding the potential neuroprotective or neural suppressive effects of anesthesia as well as ways to mitigate these concerns are discussed later in this review (see Considerations for Researchers). Similarly, many (but not all) pre-clinical TBI models require a surgical procedure prior to the injury itself. In CCI, this is a fairly large craniectomy, whereas, in fluid percussion injury (FPI), a smaller craniectomy and placement of the leur-loc device occur. As with anesthesia, use of sham controls mitigates these concerns. Experimental design choices surrounding craniectomy are discussed in additional detail below. A final issue with all pre-clinical models is that each produces one or more aspect of clinical TBI consequences (102), but none perfectly mimic the entire human condition; researchers must carefully consider the outcomes of interest in their study and which TBI model(s) best produce deficits in the chosen domains.

It is also worth noting that some of the key assets of pre-clinical models have associated limitations as well. For example, experimental TBI studies are often conducted with unprecedented control over sample characteristics that would be impossible in clinical trials. This control reduces the possible confounding effects of test subject characteristics (e.g., genotype), environmental conditions (e.g., light cycle; diet), and other factors (e.g., injury location) on study outcomes. However, since human TBI populations are characterized by variability (147), the high-degree of control in pre-clinical models may limit generalizability and slow translation. Commonly, study subjects are comprised of only one sex of animals, most often male, and a narrow age range. However, NIH has recently required that sex be formally considered as a biological variable in new grant applications. Diversification of pre-clinical samples or replication of findings in different sexes, ages, and species of animals strengthens available evidence. Recently, there has been a trend toward diversifying TBI samples when possible, although doing so is associated with increased costs.

### Limitations Specific to CCI

Limitations specific to the CCI model include mechanical variation, wear on the device, and limited diffuse effects. Discussion of how experimental design and regular maintenance can help mitigate these concerns is provided in the following section. A primary limitation surrounding the CCI device itself, which tends to be more complex than other models with respect to its mechanical parts. Moreover, the nature of the devices makes some vibration and variation inevitable, which can contribute to change over time of the relationship between the injury parameters and outcome variables. This variability is due in part to the breakdown of wearable materials that comprise the pneumatic model (e.g., seals) and wear due to friction on electromagnetic models; maintenance concerns are discussed later in this review. Another limitation of CCI is that, while it models some aspects of human TBI well, it cannot capture the full breadth of consequences seen in patients. Moreover, while CCI is an excellent model of TBI with contusion, not all human TBIs are characterized by contusion. Notably, while some diffuse effects of CCI have been reported, they are more limited than with other models (e.g., CHI; blast; FPI).

## Ongoing CCI Research: Considerations for Researchers and Future Directions

### Considerations for Researchers

To promote the highest quality CCI research possible, and address some of the aforementioned limitations, considerations pertaining to experimental design choices for CCI researchers are provided below. The discussion covers selection of the test animal, decisions surrounding the actual induction of injury (e.g., depth; dwell time, and other experimental choices). Following this discussion, the role of pilot work will be addressed more broadly.

#### Selection of Test Animal

Test animal selection should be based on the study goals, keeping in mind animal welfare goals to reduce, replace, and refine animal models and using the least sentient animal possible to adequately address the research question(s). For example, assessing the relationship between injury biomechanics and outcomes may require use of a primate or porcine brain, which are closer in size to human brains than small mammal models. It is also important to consider practical aspects surrounding the test animal, such as the housing, enrichment, and husbandry requirements, equipment required for behavioral testing, and cost. Finally, ethical considerations for working with various test animals should also be considered; researchers should review the *Guide for Care & Use of Laboratory Animals* and consult their Institutional Animal care and Use Committee (IACUC) for additional guidance.

#### Decisions Surrounding Injury Parameters

Beyond the decision to use CCI as the TBI induction technique, there are many additional choices regarding the injury (e.g., depth; tip characteristics; velocity). CCI has been used in a large number of studies, each with different experimental goals; consequently, the CCI literature is characterized by diversity in the specific injury parameters with little standardization. Overall, injury parameters typically scale up with the size of the test animal’s brain and desired injury severity. When making decisions surrounding injury parameters, researchers are encouraged to carefully consider the goals of their study, published evidence, and, whenever possible, pilot data. Considerations for the number and location of injury as well as the characteristics of the impactor tip (e.g., size, shape, surface material) will be described below.

##### Impact Location and Number

Commonly, a single injury CCI model is used, though repeated injury models have been published (29, 33, 66). In the authors’ laboratory, the preferred injury location for rats and mice is on the cortical tissue of the right hemisphere. Specifically, the craniectomy is centered between lambda and bregma so that the center of the impact tip is AP − 4 mm, R + 4 mm (rats) and AP − 3 mm, R + 3 mm (mice). The rationale to use a parasagittal injury site is that crossing the sagittal suture (i.e., midline CCI) is associated with increased bleeding (23). Researchers can adjust the location and number of impact(s) to best meet the needs of their study. Some researchers have elected to use bilateral craniectomies in an effort to promote lateral movement of tissue while studying the effects of two contusions (148, 149). For example, one study found that when animals received bilateral mild-injuries, spaced a week apart, there was damage to myelin within the corpus callosum at 60 days post-injury that was not present after a single contusion (81).

##### Impactor Characteristics: Size, Shape, Depth, Velocity, Dwell Time, Angle, and Composition

Some of the most important considerations for CCI research surround the impactor tip, including tip composition, surface material, tip geometry, angle, and dwell time. Depending on the device’s commercial supplier, a selection of tips (of varying size and composition) may be available off-the-shelf or as a custom order. Researchers can also make after-market modifications to their tips to meet the goals of the study. Notably, the are 8 pre-clinical CDEs related to the impactor tip including impactor angle, impactor angle measurement, impactor tip shape, impactor tip rigidity, impactor depth setting, impactor dwell time, impactor velocity, and surface material (47).

Tip size is largely dependent upon the test animal, with the tip diameter generally scaling up with the size of the brain (Table 2). For instance, 3-mm tips are commonly used for mice and 15-mm tips for pigs, with intermediate tip sizes used for rats (5–6 mm), ferrets (10 mm), and non-human primates (10 mm). Moreover, within an animal model, slightly larger tips may be used to produce more severe injury, though more commonly injury severity is adjusted with increasing impact depth.

The injury depth depends on the zero-point used when setting the desired depth. Notably, there is a lack of standardization across labs with some labs zeroing the tip to the skull, other labs zeroing the tip to the brain tissue (which occasionally herniates slightly after craniectomy), and many groups not specifying in publications which zero point was used. The frame of reference chosen when zeroing the tip should be noted in publications. Importantly, the zero point should be determined while the CCI device is statically pressurized (pneumatic) or energized (electromechanical) to minimize overshoot from the set level of impact depth.

Tip shape is commonly spherical or beveled flat. In the early ferret models (21, 22) the tips were spherical. Published CCI studies verify that round tips are still used (150, 151); however, beveled flat tips have become much more common in recent years (24, 51, 123, 152–154). While the emphasis on beveled tips occurs in many animal models of CCI, it is especially true when the test animals are mice. However, this seems to be largely due to convention, as little published empirical evidence surrounding the effects of tip shape. One notable study in C57BL6 mice found that, compared to round tips, beveled flat tips resulted in a greater extent of both neuronal loss and cortical hemorrhaging (155).

As with tip size, depth of injury tends to be scaled up with the size of the test animal (Table 2) as well as the desired injury severity; however, it is important to note that within a single test animal, and injury severity, variation in impact depth occurs. Still, scaling impact depth to adjust injury level is common in the literature. One study tested four different injury depths (1.5, 1.75, 2.0, and 2.5 mm) on rat pups (7 and 17 days old, with the older animals receiving greater impact depth); in this study, worsening MWM performance and histological changes were associated with increasing depth of tissue deformation (119). An electromagnetic CCI study found that the size of the lesion progressively increased with the impact depth (1.5, 2.0, and 2.5 mm), while holding the tip size, dwell time, and velocity constant (3.5 mm, 0.1 s, 5.25 m/s, respectively); progressive increase in cognitive deficits, but not emotional deficits, was also reported with increasing depth (63).

Little empirical evidence surrounding how adjusting the velocity affects outcomes. Generally, the consensus is that increasing velocity has some effect on TBI outcomes and the relative contribution of contact velocity to injury outcomes is similar in both midline and lateral CCI (156). Notably, beyond 3 m/s, depth seems to be a greater determinant of injury severity (156). While across-study variation in velocity exists in published literature, other methodological differences confound interpretation of how speed affects outcomes of CCI. Additional evidence that depth is more important than velocity in affecting injury severity comes from a finite element simulation of CCI (157). In this study, increasing the velocity by 100% was associated with increased maximum principal strains (MPS) of ~9–26.7% depending on brain region (9% in thalamus; 19.5% in deep cortex; 20.2% in hippocampus; and 26.7% in superior cortex). Notably, a 50% increase in velocity was associated with more modest increases in MPS ranging from 1.2 to 13.7% (1.2% in thalamus; 8.5% in hippocampus; 12.2% in deep cortex; and 13.7% in superior cortex). Conversely, a 35% increase in impact depth (from 2.0 to 2.7 mm) was associated with increased MPS ranging from 16.6 to 35.7% (16.6% in deep cortex; 25.8% in hippocampus; 26.1% in superior cortex; and 35.7% in thalamus). Thus, larger alterations in intracranial responses occur when depth rather than velocity is scaled up (157). Still, researchers should use a velocity sensor to ensure that the velocity is consistent with the desired setting; moreover, researchers should report the velocity of impact as part of the TBI pre-clinical CDEs (47).

Other less well-studied considerations regarding injury parameters include the orientation of the tip relative to the brain tissue or exposed skull, the composition of the tip surface material, and the dwell time. With respect to tip surface material, most studies use metal tips. However, this need not be the case, and some researchers have modified the impactor tip material to better meet the study goals (33), as described above. All injury parameter decisions should be informed by a thorough review of the literature and, whenever possible, pilot data. Also, it is important to remember that in order to ensure transparency in research and promote replication of findings, reporting of injury parameters should be done in accordance with the TBI pre-clinical CDE proposed by the NINDS (47).

##### Injury Severity

It is important to acknowledge that while the injury parameters (e.g., depth, tip size, velocity) are manipulated to control the injury severity, there is no standardization across labs with respect to what constitutes a mild, moderate, or severe injury. Moreover, depending on the outcome(s) of interest to the researcher the same CCI parameters can result in little-to-no deficit on one outcome and severe deficit on another. Thus, it is important for researchers to use pilot testing to determine the impact parameters needed to produce the extent of deficits desired pathology relevant to the clinical level of injury of scientific interest. Commonly, extent of injury is made more severe by increasing one or more of the following parameters: tip diameter, depth, and velocity. Typically, tip diameter and depth are the primary contributors to injury severity because early characterization of the model found that, beyond 3 m/s, depth seems to be a greater determinant of injury severity (156). It should be noted that different clinically relevant outcomes may require different CCI impact parameters. For example, an impact velocity of 4 m/s, 2.5 mm depth, and a 50-ms dwell time using a 5-mm spherical tip has been reported to produce a significant motor deficit, but no special memory deficit at 2 weeks post-injury (65).

#### Other Important Choices

##### Anesthesia

Choices surrounding anesthesia have the potential to impact experimental outcomes. Indeed, the NINDS recognizes the importance of reporting details about anesthesia as core CDEs for all experimental TBI models, including the type, route, and duration of anesthesia (47). Careful selection and reporting of anesthesia details is important because some agents may confer neuroprotection while others may cause neural suppression. Furthermore, some post-surgical analgesics promote neural suppression and may impair performance on behavioral testing and potentially mask benefits of treatment.

Isoflurane has neuroprotective properties (158) and has been associated with less hippocampal damage and fewer behavioral deficits when compared to fentanyl-anesthetized animals (159). Still, isoflurane remains a popular and widely used anesthetic for CCI research as well as other models of experimental brain injury. Ketamine is another agent that has been found to have neuroprotective properties attributed to antagonism of *N*-methyl-d-aspartate (NMDA) receptors (160). Similarly, halothane has been found to be neuroprotective after CCI (161). Conversely, fentanyl contributes to neural suppression (162).

Notably, the confounding effects of anesthesia are mitigated when sham animals are used as controls instead of naïve animals. There are strategies to reduce the effects of anesthesia. Some researchers perform the craniectomy while the animal is anesthetized but then discontinue anesthesia and perform impact at the time of emergence of the toe-pinch response (119). Less commonly, a closed head CCI study was approved that forgoes anesthesia altogether. One notable study used a cone-shaped bag to comfortably restrain the animals and position the head for the impact (33). For researchers who prefer to use anesthesia throughout the surgery, the effects of anesthesia can be reduced using proper dosing to avoid under- or over-sedation; standard assessments, such as the toe-pinch and other tests of reflexes can be used to assess the level of sedation in unparalyzed animals.

##### Craniectomy

There are several aspects of the craniectomy preceding CCI that can influence the study findings (80), including the method used to produce the craniectomy (163), location of the craniectomy (23), number of craniectomies (148, 149), and whether the bone flap is replaced (164, 165) or the craniectomy artificially sealed. One study found that regardless of whether the craniectomy was produced using either a manual trephine or an electric drill there were pathophysiological changes, including inflammation and evidence of a lesion, when compared to naïve test animals; notably, in this study the extent of pathophysiological changes were greatest when a drill was used (163). Despite this evidence, most researchers prefer using a drill to a trephine because it is a convenient and efficient method. Obviously, researchers using CCI to study CHI do not face these concerns. Those using the invasive CCI model can reduce the deleterious effects of craniectomy through proficiency in the procedure and careful removal of the bone flap. During the procedure, researchers should monitor for bleeding, herniation, and dura breech; these complications may warrant exclusion of some animals from the final sample. Another way to minimize the deleterious effects of craniectomy is to limit heat production during drilling; one strategy is to use a syringe to apply sterile 0.9% saline solution during the procedure, especially if a drill is used. Overall, consistent and clean craniectomy is fundamental in ensuring high-quality CCI research.

##### Experimental Endpoints

One of the most fundamental choices surrounding all pre-clinical models of TBI is the choice of histopathological and functional outcome and the details surrounding how variables are measured. Choice of outcomes should be based off study goals, and informed by a thorough review of the literature. Specifics regarding the measurement of each outcome should, whenever possible, be based off pilot testing. Beyond choice of a particular behavioral test (e.g., MWM), which subtasks to include (e.g., hidden platform task, visible platform task, probe trial), and additional details (e.g., number of trials; inter-trial interval) must be considered. A review of the techniques for studying the physiologic and behavioral consequences after experimental brain injury, including research using the CCI model, has been described in detail elsewhere (101, 102).

#### Comparison of CCI to Other Pre-Clinical Neurotrauma Models

There are several experimental TBI research available to researchers, most notably CCI, FPI, and blast-induced TBI (bTBI). Each model mimics certain histopathological and functional outcomes of clinical TBI, but no one model is able to fully represent the human condition. Researchers should carefully consider the outcomes of interest when choosing a model. CCI is a focal model of injury with limited diffuse effects; this is in contrast to FPI and bTBI. As described in a recent review (102), long-term outcomes of CCI include gross histological changes, ventricular enlargement, apoptosis, necrosis, axonal injury, cerebrovascular pathology, inflammation, and neurogenesis; CCI also causes several long-term deficits in functional status, including overall neurological status, motor function, cognitive status, and frontal lobe function (102). In contrast to CCI, FPI has not been associated with long-term changes in gross histology, inflammation, or frontal lobe function; however, unlike CCI, FPI causes long-term autophagy and stress-related outcomes (102). In contrast to CCI and FPI, bTBI is less well-characterized; bTBI has been found to cause long-term ventricular enlargement, apoptosis, axonal injury, cerebrovascular pathology, inflammation, cognitive dysfunction, and stress-related outcomes (102).

### The Importance of Regular Maintenance of CCI Devices

As is true for all laboratory equipment, high-quality CCI research depends on a well-maintained CCI device and related equipment. Prior to experiments, researchers should test the CCI device and ensure that it is in proper working order and that the piston fires freely. It is important to perform preventative maintenance, monitor the device’s performance over time, and make repairs accordingly to avoid workflow interruptions and reduced data quality. Researchers should consult with the device manufacturer surrounding the recommended maintenance plan.

Beyond manufacturer recommendations, considerations for the device’s parts, their function, and their structure can inform the maintenance routine. For example, pneumatic seals will wear over time and may result in leaks and subsequent failure of the chamber to fully pressurize, which could affect performance, including reducing maximum speed, or even prevent the piston from firing altogether. Similarly, in devices where the piston’s motion is stopped by a soft surface (e.g., rubber), there will be wear over time. Changes in the pressure to velocity calibration curve and new mechanical noises are symptoms of a malfunctioning device. Depending on the device, these parts will need to be replaced periodically; seals may also require periodic lubrication. Leaks in the hoses that deliver air to the cylinder can also occur as the polymer(s) that comprise this component degrade. It is also important to maintain the air compressor (e.g., oil level and tank moisture level), and speed-sensor calibration (e.g., periodically; after long periods of disuse; when other maintenance is performed that results in repositioning of the sensors). Electromagnetic CCI device maintenance includes replacing parts that wear due to friction as the parts move past one another and ensuring electrical components remain in proper working order.

Since much of the wear is use-related, laboratories should tailor their maintenance schedules of the CCI device based on their specific use and needs. Periodic injuring of test animals to evaluate for a marker of injury severity (e.g., hematoxylin & eosin; cresyl staining), combined with accurate records surrounding how and when the device is used be used to determine how many hits the device can deliver before significant variation occurs so the researcher can ensure replacement parts are available. Similarly, high-speed videography is a valuable tool that researchers can use to evaluate the approximate extent of mechanical variation and monitor for changes over time. Lastly, changes in brain edema (wet/dry weights) can be used to monitor injury intensity over time.

### Guidelines for Publication Including Common Data Elements

The final step in completing high-quality experimental TBI research surrounds publication of findings in such a way that studies can be replicated. To assist in this effort, the NINDS has published a list of CDEs for pre-clinical TBI research studies (47). Many of the CDEs are common to all pre-clinical TBI studies, regardless of the model used, such as characteristics of the test animals (e.g., species; strain; commercial supplier; sex; age; weight), details regarding animal treatment (e.g., housing; husbandry; euthanasia method), and techniques for assessing outcomes (e.g., specific tests/measures used; timing of outcome assessments). The guidelines also include specific CDEs for each injury model. Details regarding CDEs specific to CCI were discussed throughout the text of this review, and will be summarized here. Briefly, CCI studies should report: craniectomy size, impactor tip details (e.g., shape; size; rigidity; angle); and other injury parameters (e.g., dwell time; velocity; depth). Not only do these CDEs represent a guide for researchers when publishing their data, they may also be useful to researchers in designing CCI (and other experimental TBI) studies.

## Remaining Knowledge Gaps and Future Directions

As contemporary researchers build on the rich history of CCI, there remain gaps in the TBI knowledge base and limitations with the model. Future directions for CCI research include making incremental improvements in the device, enhancing control over potential confounders. In the future, findings will be disseminated in a way that promotes replication, by participating in the NINDS’s pre-clinical TBI CDEs. Efforts to improve the model should include reducing the mechanical variability of the device and achieving closer tolerances. Ongoing areas of inquiry include application of CCI to more genetically modified test subjects and further evaluation of the genomic, epigenomic, proteomic, and microbiomic factors underlying injury recovery.

## Conclusion

Over 100 years after the first pre-clinical TBI research, experimental models remain a mainstay. Since its development in the 1980s, the CCI model has become one of the most widely used pre-clinical TBI models. Early investigations using this model sought to determine the biomechanical properties of brain tissue exposed to direct mechanical deformation, such as those associated with automotive crashes. CCI has since been applied to evaluate the consequences of open and closed head injury, test novel therapies, as well as explore the molecular-genomic factors relevant to TBI symptom- and recovery-profiles.

Controlled cortical impact has several notable strengths, including a high-degree of control over injury parameters (e.g., velocity; depth; duration; and site of impact). The CCI model is clinically relevant in that it produces morphologic and cerebrovascular injury responses similar to aspects of human TBI and can be used to model injury across the lifespan. Commonly observed consequences of CCI include graded histologic and axonal derangements, disruption of the blood–brain barrier, subdural and intraparenchymal hematoma, edema, inflammation, and alterations in cerebral blood flow. Many functional deficits have been observed after CCI as measured using standardized behavioral tests. While CCI is characterized by focal loading with contusion, diffuse effects have also been reported.

Limitations of CCI include the non-clinically relevant craniotomy, use of anesthesia, and potential mechanical variation. Inclusion of surgical sham control groups and thoughtful study design can temper these concerns to some extent. Future directions for CCI research include continuing to make technical improvements in CCI devices and further expanding the CCI knowledge base. In publishing the results of CCI studies (and other pre-clinical TBI research), CDEs should be reported in accordance with the guidelines set by NINDS for pre-clinical TBI research.

## Author Contributions

NO and CD co-wrote and edited the manuscript. NO produced the figures.

## Conflict of Interest Statement

The authors declare that the research was conducted in the absence of any commercial or financial relationships that could be construed as a potential conflict of interest.
